# Spatiotemporal variability of surface water quality in tropical agriculture-dominated catchments: insights from water quality indices

**DOI:** 10.1038/s41598-025-27066-x

**Published:** 2025-12-02

**Authors:** Alex Saturday, Mathew Herrnegger, Susan Kangume, Gabriel Stecher

**Affiliations:** 1 Faculty of Agriculture and Environmental Sciences, Kabale University, Kabale, Uganda; 2 Institute of Hydrology and Water Management, Department of Landscape, Water and Infrastructure, BOKU University, Muthgasse 18, Vienna, 1190 Austria

**Keywords:** Surface water quality, Weighted arithmetic water quality index, Comprehensive pollution index, Principal component analysis, Maziba catchment, uganda, Ecology, Ecology, Environmental sciences, Hydrology, Water resources

## Abstract

Surface water quality in tropical, agriculture-dominated catchments faces intense pressure from human activities, yet comprehensive, index-based assessments for these regions remain limited. This study aimed to use an index-based assessment to examine the spatial and temporal changes in water quality within the Maziba catchment in southwestern Uganda, characterised by increasing land-use pressures. Monthly surface water samples were collected from 16 stations between July 2023 and June 2024 to analyse physicochemical parameters. The study employed the Weighted Arithmetic Water Quality Index (WAWQI) for assessing drinking water suitability, the Comprehensive Pollution Index (CPI) for evaluating aquatic ecosystem health, and a new combined risk framework to deliver an integrated, stakeholder-oriented assessment. WAWQI results ranged from “good” to “unfit for consumption”, with 69% of stations classified as “poor” to “unfit”. CPI indicated “slight pollution” on average. Notably, the integrated risk assessment did not classify any stations as “Low Risk”, while most were classified as “High Risk” (50.0%) or “Severe Risk” (18.8%). Human activities and seasonal changes have a significant impact on water quality deterioration in the Maziba catchment. The simultaneous decline in water suitability for drinking and ecosystem health underscores the need for integrated management strategies that target both diffuse and point-source pollution to protect public health and aquatic ecosystems.

## Introduction

 Surface water quality in tropical agricultural zones is deteriorating under intense anthropogenic pressure, a trend that jeopardises public health, ecological stability, and sustainable development. The degradation of these water resources caused by nutrient loading, agricultural chemicals runoff, and soil erosion is concerning because these regions are crucial for global food security^1,2^. These impacts are exacerbated by tropical climate patterns, where heavy rainfall increases pollutant transport, and dry seasons concentrate contaminants in reduced water volumes^3–5^. The public health consequences of surface water degradation are extensive on a global scale. While sources like rivers and dams supply nearly one-third of the world’s drinking water, a minimum of two billion people consume water contaminated with faeces^6,7^.

Water quality in agricultural catchments is influenced by a complex interplay of natural processes and human activities^3,6,8^. In tropical regions, the combined effects of agriculture, urbanisation, population growth, and industrialisation are primary drivers of water contamination^1,9,10^. For instance, inadequate wastewater treatment and extensive fertiliser application lead to nutrient enrichment, fostering excessive algal growth, dissolved oxygen (DO) depletion, and foul odours from anaerobic microbial breakdown^6^. These factors harm human health and ecosystems by introducing pollution that surpasses the natural capacity for purification^11^. While natural self-purification processes driven by bacterial metabolic responses, can break down organic materials when sufficient DO is present^12^, intensive agricultural practices and urbanisation frequently overwhelm these mechanisms. For instance, agricultural intensification and urbanisation have contributed to a decline in water quality in the Niger River in Bamako, Mali, representing a widespread environmental issue in tropical regions^13,14^.

Given the increasing pressures on freshwater resources, water quality evaluation has gained importance. Water Quality Indices (WQIs) offer a practical method for this evaluation by transforming multiple water quality parameters into a single numerical value, simplifying interpretation and informing decision-making^15^. Since their initial proposal by Horton (1965)^16^ and Brown et al. (1970)^17^, a variety of WQIs have been developed for different applications and regions. These include the US National Sanitation Foundation Water Quality Index (NSFWQI), Canadian Council of Ministers of the Environment Water Quality Index (CCMEWQI), British Columbia Water Quality Index (BCWQI), Weighted Arithmetic Water Quality Index (WAWQI), Oregon Water Quality Index (OWQI), or Comprehensive Pollution Index (CPI)^18,19^. Each index employs specific parameters, weighting schemes, and aggregation methods tailored to its respective assessment objectives. Beyond assessing current water quality, these indices are valuable tools for trend analysis and supporting environmental management decisions^14^.

The WAWQI and CPI are helpful as they incorporate parameters relevant to both human consumption and ecological health^20^. The WAWQI assesses the suitability of drinking water, incorporating parameters such as nutrients, dissolved oxygen, and bacterial indicators that reflect human health risks. CPI can evaluate ecological conditions, as it effectively identifies waters with multiple stressors and can distinguish between slight and severe pollution levels^21,22^.

The Maziba catchment, a transboundary system between Uganda and Rwanda, serves as a prototypical example for the mountainous region bordering the Democratic Republic of Congo (DRC). It provides surface water for domestic, agricultural, and hydropower needs. At the same time, its landscape is characterised by the region’s common challenges, including steep topography, high population density, and intensifying land-use pressures. Human activities, including agriculture on steep slopes, deforestation, improper waste disposal, and urbanisation, increasingly threaten water quality through accelerated erosion and pollutant transport. Untreated wastewater from Kabale town is discharged chiefly directly into the surface water system. These practices introduce diffuse pollutants, such as sediments from erosion, excess nutrients, and chemical contaminants, which degrade the quality of surface water. Despite the reliance of local communities on surface water, comprehensive data on its current water quality status is lacking. Conventional water quality assessments in the Maziba and other similar tropical catchments are often fragmented^23^, focusing on individual parameters that fail to provide a holistic picture. Data from official authorities are often difficult to access or unavailable, and few published papers detail the distribution of water quality or provide comprehensive datasets^24–27^.

Given the multiple pollution sources in the Maziba catchment, an integrated assessment approach is essential. The Water Quality Index (WQI) offers this holistic perspective by aggregating multiple parameters into a single, interpretable value that reflects overall water quality^28^. While studies have demonstrated the effectiveness of WQIs in assessing water quality in agricultural and mixed-use catchments globally^29,30^, the application to the African tropics and catchments like the Maziba remains scarce. This study evaluates how multiple anthropogenic pressures and seasonal dynamics influence water quality in the Maziba catchment through an integrated assessment. It characterises the spatiotemporal variability of its physicochemical parameters, uses multivariate statistical analysis to identify the principal drivers of water quality changes, and applies the WAWQI and CPI indices to produce a holistic evaluation of its suitability for human and ecological use. These indices are then integrated into a combined risk framework to create a single, stakeholder-oriented classification for management purposes. The complete dataset from this research has also been made publicly available, providing a comprehensive water quality dataset from the African tropics that is rarely published^31^. This research supports Sustainable Development Goal (SDG) 6 (Clean Water and Sanitation) by evaluating water quality threats and pinpointing pollution sources that endanger safe water access in tropical agricultural areas. The study also advances SDG 15 (Life on Land) by examining ecosystem health impacts and safeguarding aquatic biodiversity.

## Materials and methods

### Study area

**Fig. 1:**
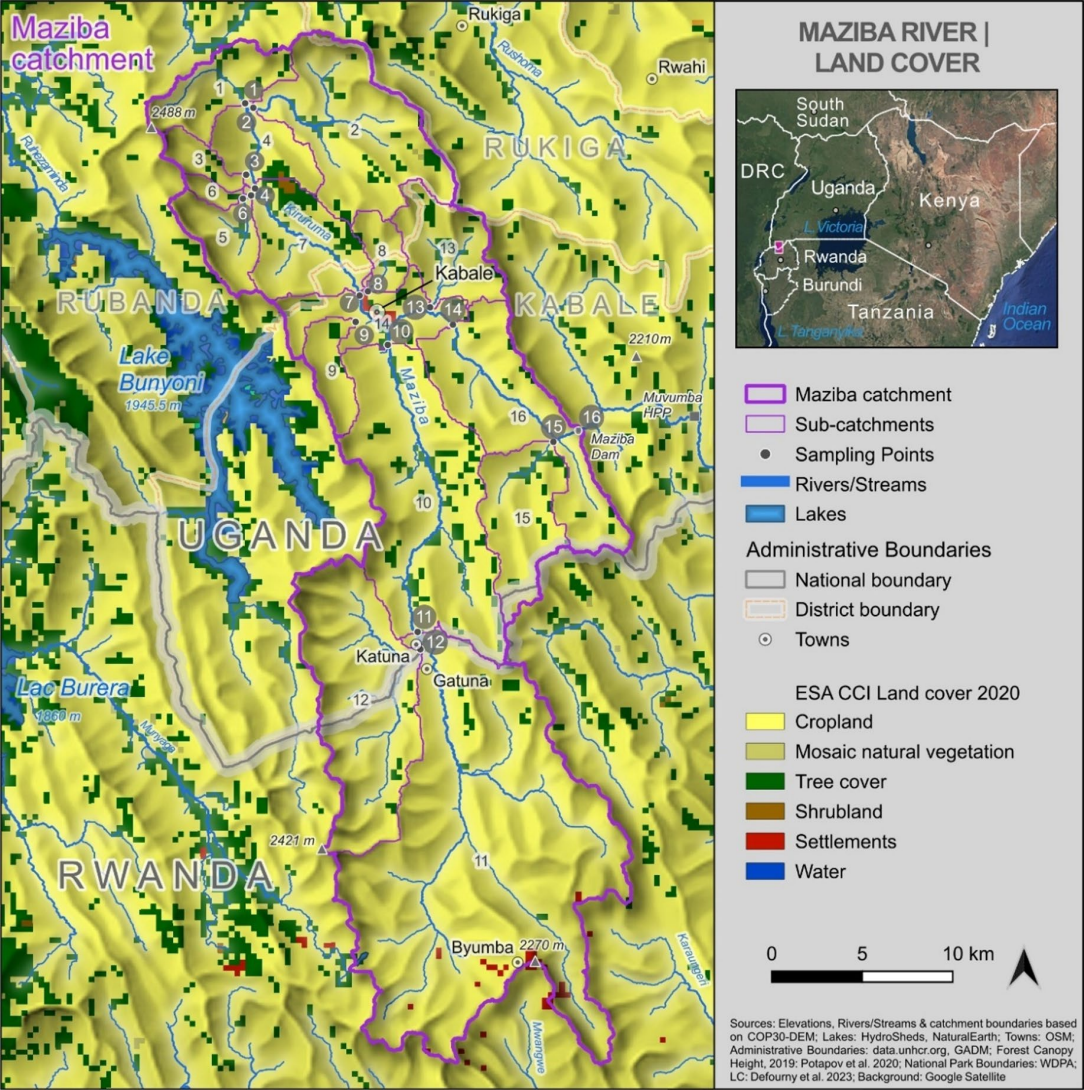
Map of the Maziba catchment showing the 16 water quality sampling stations and land cover distribution. The catchment extends from southwestern Uganda into Rwanda, with elevation ranging from around 1,760 m at Maziba Dam to 2,488 m in the northeast. Land cover is dominated by cropland (yellow) with limited tree cover (dark green). Numbers 1–16 with a dark circle/background indicate sampling stations, while numbers with a white background represent sub-catchments listed in Table 1. The Maziba drains towards the Kagera river.

The Maziba catchment, located in southwestern Uganda (29.9°–30.1°E, 1.1°–1.6°S), extends into Rwanda. The catchment covers an area of 722 km² with elevations ranging from 1,757.7 m at Maziba Dam (1.31°S, 30.09°E), which serves as the outlet, to 2,488 m in the north-eastern highlands (Fig. 1). The catchment can be divided into three sections: the Upper Maziba (covering parts of Rubanda and Kabale Districts), the Middle Maziba (covering parts of Rukiga and Ntungamo Districts), and the Lower Maziba (situated within Ntungamo District). The catchment is dominated by subsistence agriculture (~ 85% of livelihoods), with major crops including maize, Irish and sweet potatoes, bananas, beans, and vegetables for subsistence, while tobacco, coffee, fruits, pyrethrum, sorghum, wheat, and millet serve as the primary cash crops. The region’s steep topography, combined with intensive agricultural land use on hillslopes, results in a high erosion potential and diverse sources of pollution. Despite these factors, the overall water quality status throughout the catchment remains poorly understood.

**Fig. 2:**
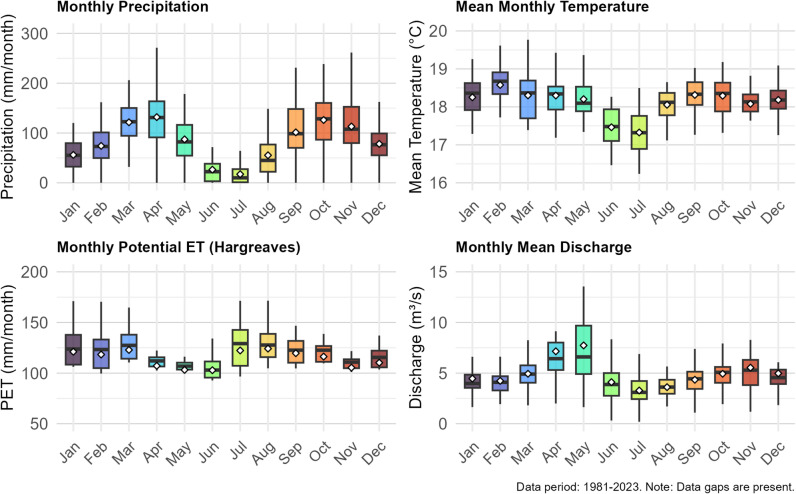
Monthly seasonality of climate and hydrological parameters for the Maziba catchment, Uganda. Daily meteorological data (precipitation and temperature) were obtained from Kabale Meteorological Station, while daily discharge measurements were taken downstream of Maziba Dam. Potential evapotranspiration (PET) was calculated using the Hargreaves method. Boxplots display the median (horizontal line), interquartile range (box), and whiskers extending to 1.5 times the interquartile range. White diamonds indicate the mean. Monthly precipitation and PET are shown as sums, while mean monthly temperature and discharge represent monthly averages of daily values. Data spans 1981–2023, with various gaps present.

The climate of the Maziba catchment, based on long-term (1981–2023) meteorological records from Kabale station and discharge data downstream from Maziba Dam, displays distinct seasonal patterns (Fig. 2). Precipitation follows a distinct bimodal pattern, with the main wet seasons typically occurring from March to May and again from September to November or December. Drier conditions typically occur from June to August and in January to February. On average, annual precipitation amounts to about 1033 mm, with totals ranging from 759 to 1225 mm. The mean annual temperature remains relatively constant, averaging 18.2 °C, with annual means between 17.3 and 18.8 °C. Potential evapotranspiration (PET), calculated using the Hargreaves method, averages around 1,441 mm, with yearly totals from 1,246 to 1,624 mm. The annual discharge generally reflects the rainfall patterns, peaking after the wet seasons and decreasing during dry periods, with an average of 4.88 m³/s and yearly means from 1.29 to 10.2 m³/s. It should be noted that the data for these annual figures vary due to differences in data completeness across parameters.

### On-site measurement, sample collection and storage

Water samples were collected monthly from 16 strategically selected sites across various watersheds of the upper Maziba sub-catchment over a 12-month period (July 2023–June 2024) (Fig. 1; Table 1). Selection criteria were based on land use intensity, population density, and accessibility. On-site measurements of physicochemical parameters, including water temperature (WT), electrical conductivity (EC), dissolved oxygen (DO), and pH, total dissolved solids (TDS), were conducted in accordance with the American Public Health Association (APHA) guidelines (2023)^32^. A water-resistant handheld pH and EC meter (HI98130) measured temperature, pH, EC and TDS, while the DO was assessed using a DO meter (PDO-519 model). An Aquafluor™ handheld fluorometer was used to measure the turbidity and Chl-a from a well-agitated sample in a cuvette, and readings were recorded after stabilisation. Water samples for laboratory analysis were collected in 1-litre plastic bottles and transported in a cool box to the Ministry of Water and Environment’s National Reference Laboratory (Entebbe) for physicochemical analysis. While in the laboratory, samples were stored at 4 °C, awaiting analysis.

**Table 1:** Characteristics of the 16 water quality sampling stations in the Maziba sub-catchment, showing station codes, names, contributing sub-catchment areas, total upstream drainage areas, elevation, and geographic coordinates.

Code	Station Name	Sub-catchment Area (km²)	Total Upstream Area (km²)	Altitude (m)	Latitude	Longitude
M1	Hakakondogoro	22.74	22.74	1839.5	−1.1521	29.9252
M2	Kakore East	61.45	61.45	1838.5	−1.1544	29.9293
M3	Mukirwa	7.38	7.38	1837.7	−1.1877	29.9257
M4	Ihanga FGC	18.45	110.02	1827.1	−1.1945	29.9300
M5	Ihanga	16.23	19.57	1832.9	−1.1980	29.9281
M6	Ihanga West	3.34	3.34	1849.9	−1.1995	29.9240
M7	Rwakaraba Bridge	32.44	162.03	1800.8	−1.2478	29.9819
M8	Lower Bugongi	9.10	9.10	1803.9	−1.2457	29.9860
M9	Brazin Forest	11.98	11.98	1803.5	−1.2609	29.9799
M10	Rugyendira	99.19	399.84	1791.3	−1.2724	29.9959
M11	Muvumba	226.22	300.65	1805.1	−1.4151	30.0105
M12	Katuna	74.43	74.43	1806.5	−1.4238	30.0119
M13	Butobere	26.15	26.15	1796.5	−1.2537	30.0169
M14	Kyanamira Bridge	25.22	634.32	1786.5	−1.2624	30.0282
M15	Kabanyonyi	32.30	32.30	1759.7	−1.3207	30.0781
M16	Maziba Dam	55.08	721.70	1757.7	−1.3152	30.0904

### Laboratory analysis

Total nitrogen (TN), total phosphorus (TP), nitrate nitrogen (NO₃⁻-N), ammonium nitrogen (NH₄⁺-N), Nitrite nitrogen (NO₂⁻-N), and soluble reactive phosphorus (SRP), Chloride (Cl^−^), Sulphates (SO₄²⁻), sodium (Na⁺), and Potassium (K⁺) were analysed in the laboratory using a discreet photometric analyser (Thermoscientific gallery plus model), following the standards set by APHA (2023)^32^. The fully automated discreet analyser provided quality repeatable water analysis results with minimal errors, thus ensuring confidence in the quality of analytical results. Total suspended solids (TSS) was determined by the standard gravimetric method in accordance with APHA (2023) standard guidelines.

### Statistical analysis

Data analyses were conducted using Statistical Package for the Social Sciences (SPSS) 27.0 and R. Before statistical analysis, the Kolmogorov–Smirnov test was employed to determine whether the data followed a normal distribution. The Kruskal–Wallis test was used for non-normally distributed data, and one-way analysis of variance (ANOVA) for normally distributed data to assess differences in the measured parameters across stations and months. The Mann-Whitney U test was used to determine whether significant differences existed in measured parameters between seasons, except for non-normal data, where an independent-samples t-test was employed. A principal component analysis (PCA) was conducted to identify the key physicochemical parameters that contribute most to variability. The Kaiser–Meyer–Olkin (KMO) and Bartlett’s tests confirmed the adequacy of the data for PCA. The KMO value of 0.760 exceeded the recommended threshold of 0.6, affirming the suitability of the data for PCA. Bartlett’s Test (χ² = 2078.072, df = 171, *p* < 0.001) yielded significant results, further validating the adequacy of the data for PCA. Spearman’s correlation coefficients were calculated to evaluate the linear relationships among water parameters. A significance level of 0.05 was applied to all tests, with a highly significant threshold set at 0.01.

### Water quality indices (WQI)

Index-based methods simplify water quality assessment by combining multiple parameters into scores that are easy to interpret, compare across sites and seasons, and support catchment management decisions.

### Weighted arithmetic water quality index (WAWQI) - drinking water suitability

The Weighted Arithmetic Water Quality Index (WAWQI) by Brown et al. (1972)^33^, as shown in Eqs. 1–4, assessed drinking water quality against World Health Organisation standards (WHO)^34^. To provide a more localised context, we used the standards set by the Uganda National Bureau of Standards (UNBS)^35^ for natural potable water as a reference. In total, thirteen physicochemical parameters (i.e., DO, pH, turbidity, EC, NH₄⁺-N, NO₃⁻-N, SRP, Cl⁻, NO₂⁻-N, Na⁺, SO₄²⁻, TH, and temperature) were employed to calculate the WAWQI. The standard values used to compute the index are presented in Table 2. The WAWQI is categorised into five classes: excellent, good, poor, extremely poor, and unfit for consumption, based on index values of 0–25, 26–50, 51–75, 76–100, and over 100, respectively (Table 3).

The WAWQI is calculated as follows:

1 $${\rm WAWQI} = \frac{\Sigma Q_n W_n}{\Sigma W_n}$$

with

Qn = quality rating of the n^th^ water quality parameter.

Wn = the unit weight of the n^th^ water quality parameter.

Qn is computed using Eq. (2).

2 $$Q_n = 100 \lfloor (\frac{V_n - V_i }{S_n - IV}\rfloor$$

with

*Vn* = the concentration value of n^th^ variable;

*IV* = the ideal value (*IV* = 0, except for DO (IV = 14.6 mg/L) and pH (IV = 7).

*Sn* = the standard permissible value for the n^th^ variable.

The calculation of unit weight (Wn) for the selected physicochemical variables is inversely proportional to the recommended standard/threshold values for the corresponding variables.

3 $$W_n = \frac{K}{S_n}$$

with

*K* = the constant of proportionality computed using Eq. 4.

4 $$K =\frac{1}{\Sigma \frac{1}{S_n}}$$

**Table 2:** Threshold values for WAWQI calculation.

Parameters	WHO/UNBS standards
1. DO (mg/L)	6
2. pH	8.5
3. Turbidity (NTU)	25
4. EC (µS/cm)	2500
5. NH₄⁺-N (mg/L)	0.2
6. NO₂⁻-N (mg/L)	3
7. NO₃⁻-N (mg/L)	13.29
8. SRP (mg/L)	2.2
9. Cl⁻ (mg/L)	250
10. Na⁺ (mg/L)	200
11. SO₄²⁻ (mg/L)	400
12. TH (mg/L)	200
13. Temperature (^O^C)	25

**Table 3:** Water quality index (WAWQI) classification scheme for drinking water quality assessment.

WAWQI	Water Quality Status
0–25	Excellent
26–50	Good
51–75	Poor
76–100	Very poor
Above 100	Unfit for human consumption

### Comprehensive pollution index (CPI) - ecosystem health

The assessment of river water quality for aquatic ecosystems and ecosystem health was conducted using the Comprehensive Pollution Index (CPI), as defined in Eqs. 5 and 6, applied by^8,36^. To account for local conditions, we used the Uganda National Bureau of Standards^35^ as a reference for natural potable water standards. Nine parameters (i.e., DO, pH, turbidity, EC, NH₄⁺-N, NO₃⁻-N, SRP, Cl⁻, and temperature) were used. All the standard values for computing the CPI are similar to those used in WAWQI Table 2, except for DO (8 mg/L), Cl⁻ (120 mg/L), and temperature (27 °C).

The CPI is calculated as follows:

5 $$\rm CPI =\frac{1}{n} \ast \Sigma\: PI$$

with

n = number of monitoring parameters or selected pollutants;

PI = the single-factor pollution index from each measured parameter (i),

i = starting number of monitoring parameters.

The single-factor pollution index (Pi) is calculated according to the following equation:

6 $${\rm PI} (\frac{V_n}{S_n})$$

with

Ci = measured concentration of parameter i in water.

Si = standard value of the ith parameter based on international standard guidelines for drinking purposes and aquatic life (CCME). Similar to the WAWQI, the CPI categorises pollution levels into five categories (Table 4).

**Table 4:** CPI-based water quality classification scheme (Ecosystem Health).

CPI range	Water quality status
0.0–0.2	No Apparent Impact
0.21–0.4	Minimal Impact
0.41–1.0	Slightly Impacted
1.01–2.0	Moderately Impacted
2.01 and above	Severely Impacted

### Combined risk assessment for communication and water resource management

To provide a holistic assessment of water resource health, a combined risk framework was developed. This approach integrates the findings from the WAWQI (drinking water suitability) and the CPI (ecosystem health) into a single, stakeholder-oriented classification that facilitates communication and management. By considering both human-use and ecological endpoints, this framework allows for a more nuanced understanding of the pressures on the water system and helps prioritise management actions.

Five risk levels were defined, classifying each station based on the combined status of its drinking water and ecosystem health indicators (Table 5). A Low Risk classification was assigned only to stations where both drinking water quality was high (WAWQI ≤ 50) and ecosystem impact was minimal (CPI ≤ 0.4). Conversely, a station was immediately classified as Severe Risk if either its drinking water was deemed unfit for consumption (WAWQI > 100) or its ecosystem was severely impacted (CPI > 2.0). Intermediate risk levels were defined to distinguish between primary threats. A Moderate Risk (Ecosystem) classification indicates stations with good drinking water quality (WAWQI ≤ 50) but where ecological health is compromised (0.4 < CPI ≤ 2.0). In contrast, a Moderate Risk (Drinking) classification highlights stations with poor drinking water quality (WAWQI > 50) but where ecosystem health remains good (CPI ≤ 0.4). Finally, a High-Risk classification was assigned to stations where both drinking water quality and ecosystem health were simultaneously degraded, representing a multi-faceted management challenge. This integrated assessment provides a basis for evaluating and communicating the complex trade-offs and combined impacts affecting the water body.

**Table 5:** Risk assessment matrix combining the weighted arithmetic water quality index (WAWQI) and the comprehensive pollution index (CPI) to classify overall water resource health.

		Ecosystem Health (CPI)		
		No Apparent/Minimal Impact (CPI ≤ 0.4)	Slightly/Moderately Impacted (0.4 < CPI ≤ 2.0)	(CPI) Severely Impacted (CPI > 2.0)
**Drinking Water (WAWQI)**	**Excellent/Good** **(WAWQI ≤ 50)**	Low Risk	Moderate Risk (Ecosystem)	Severe Risk
	**Poor/Very Poor** **(50 < WAWQI ≤ 100)**	Moderate Risk (Drinking)	High Risk	Severe Risk
	**Unfit for Consumption** **(WAWQI > 100)**	Severe Risk	Severe Risk	Severe Risk

## Results

### Spatial variability of physicochemical parameters

**Fig. 3:**
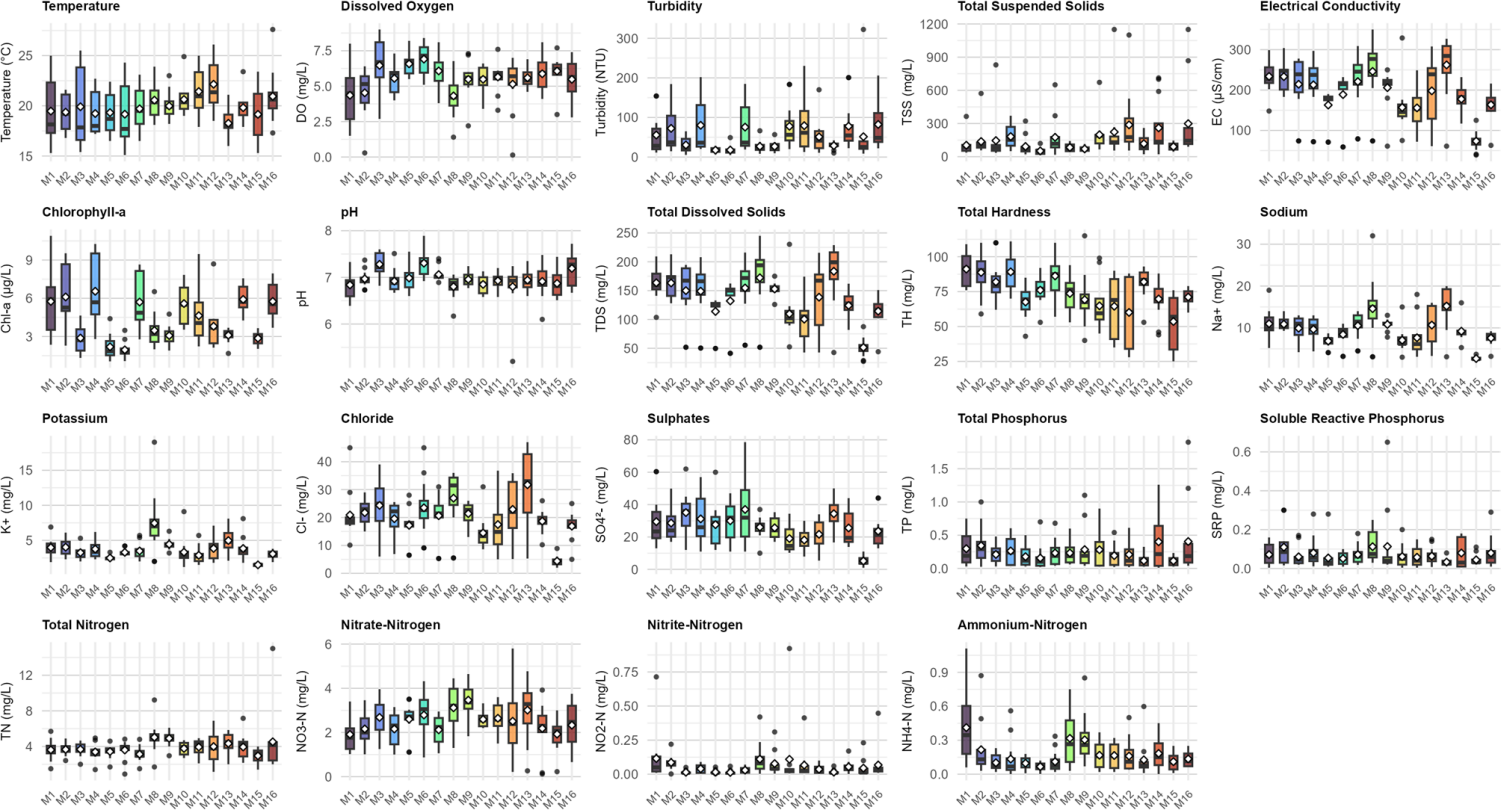
Spatial distribution of water quality parameters across 16 monitoring stations (M1-M16) in the Maziba catchment. Box plots show median (horizontal line), interquartile range (box), whiskers (1.5 × IQR), outliers (black dots), and mean values (white diamonds). Parameters are organised by category: physical parameters (temperature, dissolved oxygen, turbidity, TSS, electrical conductivity, chlorophyll-a), general chemistry (pH, TDS, total hardness), major ions (sodium, potassium, chloride, sulphates), and nutrients (phosphorus and nitrogen forms). Twelve samples were collected per station between July 2023 and June 2024, totalling *n* = 192.

Figures 3 and Appendices A1 – A3 provide a detailed summary of the variations in the measured physicochemical parameters across the studied locations. The figure shows the overall distribution of the data, including any outliers identified at particular stations. The appendices offer quantitative evaluations, including the mean values and standard deviations of the observed water quality parameters.

The highest mean temperature of 22.15 ± 2.65 °C was recorded at Katuna Station (M12), while Butobere Station (M13) showed the lowest mean temperature at 18.27 ± 1.43°c. Turbidity levels ranged from 17.44 ± 7.2 NTU at Ihanga West Station (M6) to 82.38 ± 66.69 NTU at Maziba Dam (M16). The maximum DO content was observed at Ihanga West (M6) at 6.91 ± 1.15 mg/L, whereas Lower Bugongi (M8) had the lowest at 4.31 ± 1.44 mg/L. Butobere (M13) recorded the highest mean EC value of 262.25 ± 74.99 µS/cm, while Kabanyonyi (M15) showed the lowest at 73.50 ± 23.72 µS/cm. The highest total dissolved solids (TDS) level was measured at Butobere (M13) with 172.28 ± 61.65 mg/L, and the lowest at Kabanyonyi with 51.44 ± 16.6 mg/L. The Kruskal-Wallis Test indicated statistically significant differences in temperature, DO, turbidity, EC, and TDS across the study stations (*p* < 0.05).

The Maziba Dam station (M16) recorded the highest TSS value (298.75 ± 343.81 mg/L) compared to Katuna (M12; 287.75 ± 288.54 mg/L), while Ihanga West (M6) observed the lowest mean value of 52.42 ± 30.99 mg/L. Mean chlorophyll a (Chl-a) values ranged from 1.95 ± 0.63 µg/L at Ihanga West (M6) to 6.55 ± 2.85 µg/L at the Ihanga Full Gospel Church (FGC) station (M5). The pH values varied from 6.80 ± 0.24 at Lower Bugongi (M8) to 7.30 ± 0.30 at Ihanga West (M6). Kabanyonyi (M15) also showed the lowest sodium and total hardness values, while Butobere (M13) and Hakakondogoro (M1) had the highest. Additionally, Kabanyonyi (M15) exhibited the lowest values for Na⁺, K⁺, Cl⁻, and SO₄²⁻, with Lower Bugongi (M8) registering the highest values, except for sulphates. The Kruskal-Wallis test indicated significant differences in Na⁺, K⁺, pH, Cl⁻, SO₄²⁻, total suspended solids (TSS), and total hardness (TH) values (*p* < 0.05) across the study stations.

Figure 3 and Appendix A3 also show variations in nutrient parameters across the study stations. TN values ranged from 2.95 ± 0.88 mg/L at Kabanyonyi (M15) to 4.91 ± 0.87 mg/L at Brazin Forest station (M9). The highest NO₃⁻-N concentration was at Brazin Forest (M9; 3.46 ± 0.78 mg/L), followed by Butobere (M13; 3.01 ± 1.31 mg/L), with Hakakondogoro (M1) recording the lowest value (1.91 ± 0.74 mg/L). The mean NO₂⁻-N levels were lowest at Mukirwa (M3; 0.01 ± 0.01 mg/L) and Ihanga West (M6; 0.11 ± 0.12 mg/L). At Ihanga West and Hakakondogoro, mean NH₄⁺-N levels ranged from 0.07 ± 0.04 to 0.41 ± 0.32 mg/L. For TP, values ranged from 0.16 ± 0.19 mg/L at Ihanga West (M6) to 0.40 ± 0.46 mg/L at Kyanamira (M14) and 0.40 ± 0.57 mg/L at Maziba Dam (M16). Butobere (M13) recorded the lowest SRP value of 0.03 ± 0.02 mg/L, while the highest values were observed at Kakore East (M2; 0.11 ± 0.10 mg/L), Lower Bugongi (M8; 0.11 ± 0.08 mg/L), and Brazin Forest (M9; 0.11 ± 0.19 mg/L) (Fig. 3). Statistically significant differences were observed in TN, NO₃⁻-N, NO₂⁻-N, and NH₄⁺-N (*p* < 0.05), with the exception of SRP and TP values (*p* > 0.05) across the study stations.

### Temporal variability in water quality parameters

**Fig. 4:**
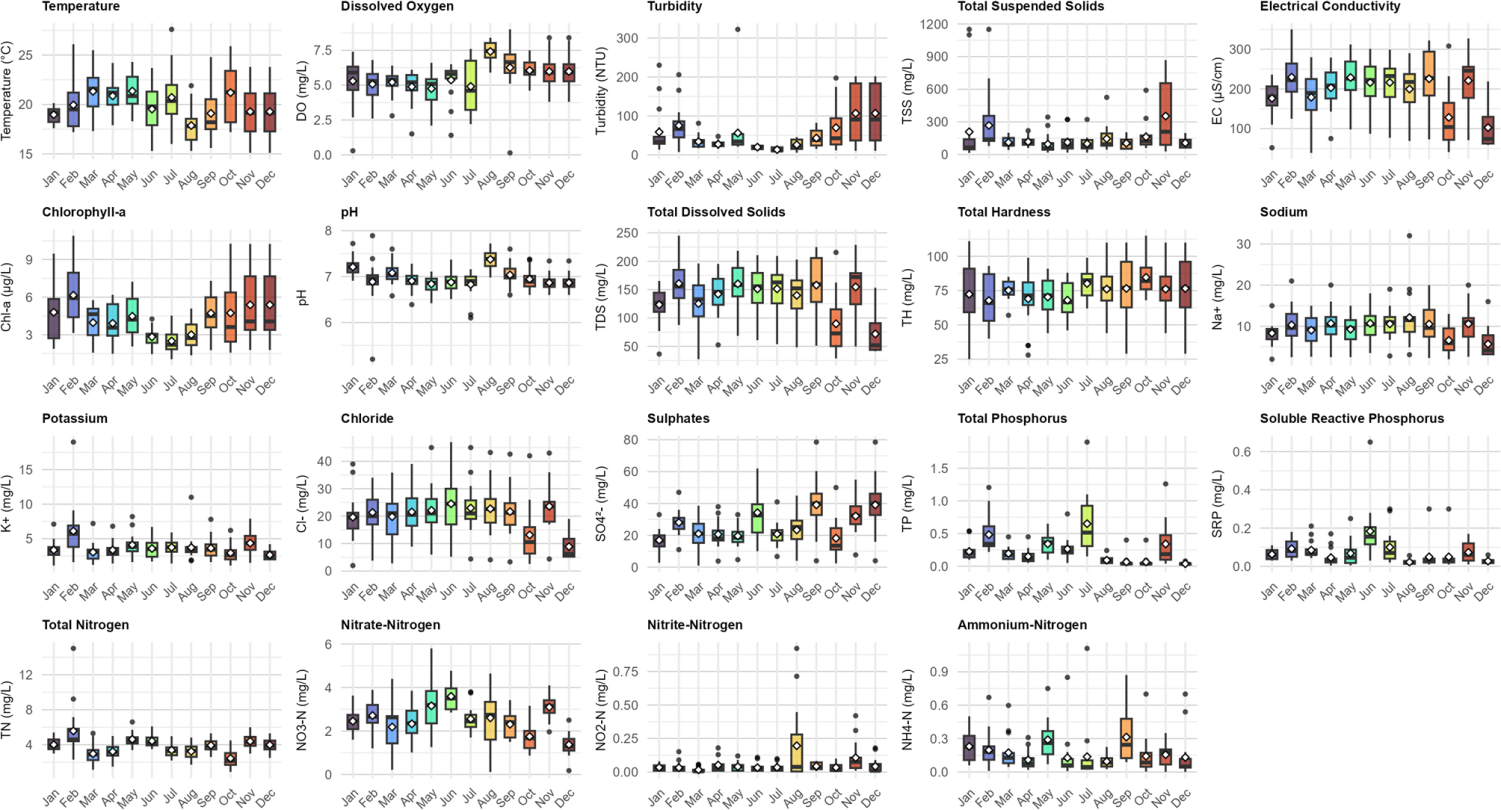
Temporal variation of water quality parameters in the Maziba sub-catchment from July 2023 to June 2024. Box plots show median (horizontal line), interquartile range (box), whiskers (1.5 × IQR), outliers (black dots), and mean values (white diamonds) for monthly samples collected from all 16 monitoring stations (*n* = 192).

Figure 4 and Appendices A4-A6 show the temporal changes in physicochemical parameters recorded during the study period, from July 2023 to June 2024. Based on these patterns, water quality parameters in the Maziba sub-catchment display complex seasonal fluctuations driven by both climate and human activities. Temperature reaches its lowest levels in August-September (~ 17–19 °C) and peaks in March-May (~ 22–23 °C). Meanwhile, dissolved oxygen exhibits an opposite pattern, with the highest levels in August and September, and the lowest from January to July, reflecting temperature-dependent oxygen solubility. Turbidity and TSS reflect the influence of the wet season; turbidity remains low from January to September (except for a small peak in February), before rising sharply from October to December. Meanwhile, TSS exhibits prominent peaks in February and November, with low levels in May. Electrical conductivity peaks in February and reaches its minimum in October and December, indicating dilution during peak rainfall periods. Chlorophyll-a concentrations stay high from January to May and September to December, with the lowest values during June to August, suggesting reduced algal productivity during the cooler dry season (Fig. 4 and Appendix A4).

Chemical parameters show varied responses: pH values are higher in January, March, and August, with lows in November and December; TDS follows EC patterns, being lower in October and December, while total hardness varies greatly, peaking in October. Major ions exhibit seasonal concentration and dilution cycles: sodium is lowest in October and December, potassium peaks in February and November but is low in March and December. Chloride remains relatively stable despite high variability and low values in October to December, and sulphates have higher levels in June, September, November, and December (Fig. 4 and Appendix A5).

Nutrient dynamics reveal particularly complex patterns: both total phosphorus and SRP peak in February, May, July, and November, with high variability during these months; total nitrogen is elevated in February, May, and June but lower in March, April, July, and October; nitrate-nitrogen peaks in May, June, and November, showing high variability in August; nitrite-nitrogen peaks in August and November; while ammonium-nitrogen is highest in May and September, with increased variability during these periods. These patterns suggest that water quality is mainly influenced by the interaction between rainfall-driven dilution and runoff processes, with the peaks observed in February and November across multiple parameters indicating periods of pollutant mobilisation during early wet season events. Additionally, the high nutrient variability during specific months indicates episodic pollution inputs, likely from agricultural activities coinciding with planting and fertilisation cycles. Statistically significant differences were observed in WT, DO, turbidity, Chl-a, pH, EC, and TDS values (*p* < 0.05) across the study months.

**Fig. 5:**
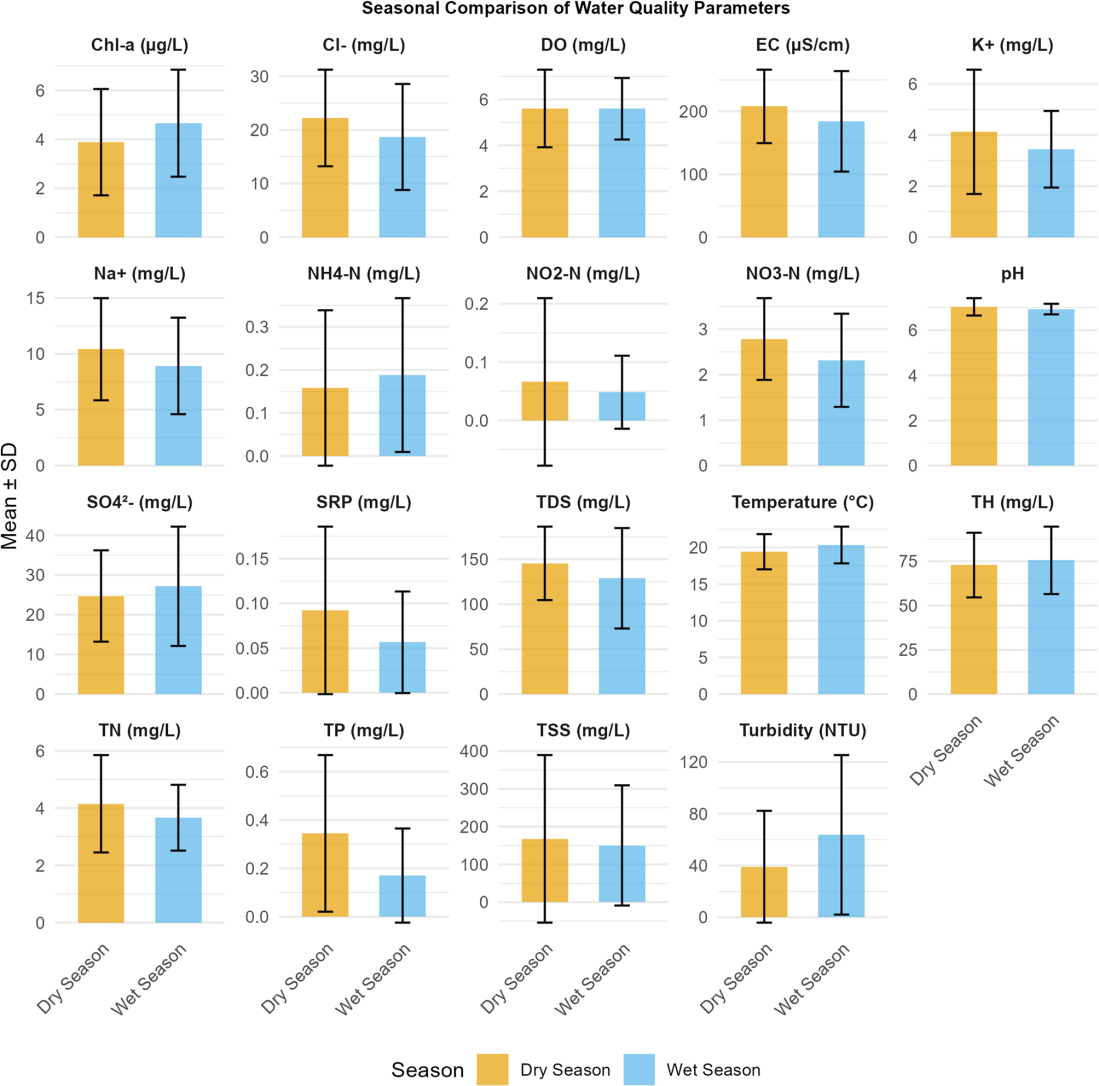
Seasonal variability of physicochemical parameters in the Maziba sub-catchment, western Uganda.

All measured parameters showed significantly higher values during the dry season (i.e., January-February and June-August) than in the wet season (September-December and March-May), with exceptions noted for Chl-a, SRP, TN, turbidity, water temperature, and NH₄⁺-N (Fig. 5). The Mann-Whitney U test indicated statistically significant differences in mean values between the dry and wet seasons, except for NO₂⁻-N, NH₄⁺-N, TH, SO₄²⁻, TSS, DO, and TN (*p* > 0.05).

### Multivariate analysis of water quality parameters

Principal Component Analysis (PCA) identified six components with eigenvalues exceeding 1, collectively explaining 72.1% of the total variance (Table 6). Table 7 summarises the primary loadings for each component. PC1, which accounted for 27.10% of the variance, was dominated by high positive loadings of EC (0.916), TDS (0.922), Na⁺ (0.886), and K⁺ (0.727), and was therefore interpreted as representing salinity and ionic strength. PC2 explained 14.80% of the variance and was strongly associated with turbidity (*r* = 0.876) and Chl-a (0.794), indicating turbidity and biological productivity. PC3 accounted for 10.79% of the variance and was linked to high loadings for SRP (0.877) and TP (0.769), indicating phosphorus enrichment. PC4 contributed 7.52% of the variance, showing positive loadings for DO (0.742) and pH (0.708), along with a negative loading for water temperature (−0.726). This was interpreted as indicating a thermal-chemical balance in the river ecosystem. PC5, which explained 6.45% of the variance, was dominated by TH, indicating hardness and ionic minerals. PC6, representing 5.47% of the variance, was mainly associated with NH₄⁺-N, reflecting ammonium/nitrogen inputs.

**Table 6:** Eigenvalues of the correlation matrix.

Component	Initial Eigenvalues			Rotation Sums of Squared Loadings		
	Total	% of Variance	Cumulative %	Total	% of Variance	Cumulative %
1	5.15	27.10	27.10	4.84	25.47	25.47
2	2.81	14.80	41.89	2.40	12.61	38.08
3	2.05	10.79	52.69	1.80	9.45	47.53
4	1.43	7.52	60.21	1.77	9.33	56.85
5	1.23	6.45	66.66	1.63	8.59	65.44
6	1.04	5.47	72.13	1.27	6.69	72.13
7	0.88	4.63	76.76			
8	0.77	4.06	80.82			
9	0.68	3.56	84.39			
10	0.56	2.96	87.35			
11	0.52	2.74	90.09			
12	0.44	2.32	92.40			
13	0.39	2.06	94.46			
14	0.30	1.60	96.06			
15	0.24	1.27	97.33			
16	0.22	1.14	98.47			
17	0.15	0.78	99.25			
18	0.12	0.64	99.89			
19	0.02	0.11	100.00			

Extraction Method: Principal Component Analysis.

**Table 7:** Summary of the main loadings for each component.

Parameters	Component					
	1	2	3	4	5	6
WT	0.012	−0.102	−0.011	−0.726	−0.272	−0.146
DO	−0.083	0.001	−0.241	**0.742**	−0.076	−0.185
Turbidity	−0.173	**0.876**	−0.032	−0.074	0.106	0.016
Chl-a	−0.100	**0.794**	0.023	−0.138	0.258	0.292
pH	−0.040	−0.243	−0.115	**0.708**	−0.054	0.012
EC	**0.916**	−0.072	0.092	−0.060	0.200	0.089
TDS	**0.922**	−0.083	0.099	−0.052	0.209	0.115
TH	0.160	−0.015	−0.045	0.016	**0.807**	−0.039
Na⁺	**0.886**	−0.091	−0.077	0.014	0.126	0.176
K⁺	**0.727**	0.190	0.105	−0.076	−0.065	0.325
NO₂⁻-N	0.148	0.052	−0.267	0.135	−0.264	**0.539**
NH₄⁺-N	0.166	0.009	0.252	−0.102	0.065	**0.733**
Cl⁻	**0.876**	−0.177	0.033	−0.026	0.073	−0.005
SO₄²⁻	0.439	0.246	0.046	0.242	0.547	−0.164
SRP	0.131	−0.042	**0.877**	−0.087	0.029	0.081
TP	0.097	0.219	**0.769**	−0.230	−0.101	−0.008
NO₃⁻-N	**0.669**	−0.107	0.235	−0.022	−0.372	−0.290
TN	**0.527**	0.375	0.318	−0.065	−0.352	−0.051
TSS	0.031	**0.758**	0.174	0.052	−0.254	−0.145

Extraction Method: Principal Component Analysis. Rotation Method: Varimax with Kaiser Normalisation.

A Spearman correlation analysis was performed to examine the relationships among the measured water quality parameters (Table 8). The results showed that SRP and TP had statistically significant positive correlations with pH, EC, TDS, Na⁺, K⁺, NH₄⁺-N, and Cl⁻ (*p* < 0.05). Additionally, Cl⁻ and SO₄²⁻ displayed strong positive correlations with EC, TDS, TH, Na⁺ and K⁺ (*p* < 0.05). Furthermore, NO₂⁻-N and NH₄⁺-N were strongly positively correlated with turbidity, Chl-a, EC, TDS, and K. Total nitrogen (TN) and NO₃⁻-N demonstrated strong positive correlations with all measured parameters except turbidity, Chl-a, and pH. Turbidity had a strong positive correlation with NO₂⁻-N, NH₄⁺-N, and TSS, while showing negative correlations with Chl-a, pH, EC, TDS, and TH (*p* < 0.05). Temperature exhibited a significant negative correlation with dissolved oxygen (DO), pH, TH, and SO₄. In addition, DO showed a negative correlation with Chl-a, pH, EC, TDS, TH, Na⁺, K⁺, NO₂⁻-N, NH₄-N, SRP, TP, and TN. TDS was positively correlated with EC, TH, Na⁺, K⁺, NO₂⁻-N, NH₄⁺-N, Cl⁻, SO₄²⁻, SRP, TP, NO₃⁻-N, TN, and TSS, but negatively correlated with DO (*p* < 0.05).

**Table 8:** Spearman’s correlation matrix between the physicochemical parameters of water in the Maziba sub-catchment.

	WT	DO	Turbidity	Chl-a	pH	EC	TDS	TH	Na^+^	K^+^	NO^2^^-^-N	NH^4^^+^-N	Cl^-^	SO^4^^2-^	SRP	TP	NO^3^^-^-N	TN	TSS
**WT**	1.000																		
**DO**	−0.311^**^	1.000																	
**Turbidity**	0.049	−0.094	1.000																
**Chl-a**	−0.029	−0.221^**^	0.813^**^	1.000															
**pH**	−0.237^**^	0.414^**^	−0.178^*^	− 0.189^**^	1.000														
**EC**	0.010	−0.206^**^	−0.169^*^	−0.048	−0.037	1.000													
**TDS**	−0.001	−0.217^**^	−0.178^*^	−0.060	−0.036	0.985^**^	1.000												
**TH**	− 0.170^*^	−0.064	−0.001	0.097	−0.004	0.270^**^	0.282^**^	1.000											
**Na** ^+^	−0.009	−0.148^*^	−0.203^**^	−0.080	0.043	0.890^**^	0.899^**^	0.264^**^	1.000										
**K** ^+^	−0.039	−0.241^**^	0.014	0.087	−0.116	0.772^**^	0.783^**^	0.113	0.774^**^	1.000									
**NO**_2_^-^-N	0.052	−0.176^*^	0.168^*^	0.222^**^	− 0.173^*^	0.209^**^	0.230^**^	0.020	0.259^**^	0.338^**^	1.000								
**NH**_4_^+^-N	0.087	−0.227^**^	0.213^**^	0.289^**^	−0.094	0.164^*^	0.189^**^	−0.105	0.123	0.259^**^	0.394^**^	1.000							
**Cl** ^-^	0.043	−0.131	−0.272^**^	−0.212^**^	0.093	0.835^**^	0.841^**^	0.223^**^	0.836^**^	0.684^**^	0.115	0.111	1.000						
**SO**_4_^2-^	−0.172^*^	0.061	0.034	0.012	0.004	0.532^**^	0.546^**^	0.303^**^	0.521^**^	0.474^**^	0.129	−0.044	0.427^**^	1.000					
**SRP**	0.122	−0.328^**^	−0.030	0.083	−0.134	0.258^**^	0.262^**^	0.020	0.172^*^	0.260^**^	0.109	0.077	0.219^**^	0.080	1.000				
**TP**	0.120	−0.414^**^	−0.045	0.123	−0.161^*^	0.298^**^	0.302^**^	−0.065	0.170^*^	0.364^**^	0.076	0.143^*^	0.243^**^	−0.053	0.670^**^	1.000			
**NO**_3_^-^-N	0.132	−0.081	−0.256^**^	−0.291^**^	−0.045	0.488^**^	0.490^**^	−0.160^*^	0.426^**^	0.452^**^	0.117	0.071	0.531^**^	0.268^**^	0.260^**^	0.284^**^	1.000		
**TN**	0.084	−0.217^**^	0.120	0.086	−0.222^**^	0.376^**^	0.382^**^	−0.128	0.309^**^	0.524^**^	0.195^**^	0.290^**^	0.351^**^	0.341^**^	0.216^**^	0.246^**^	0.644^**^	1.000	
**TSS**	0.094	−0.046	0.530^**^	0.488^**^	−0.139	−0.092	−0.110	−0.106	−0.113	0.021	0.024	−0.061	−0.150^*^	0.018	0.009	0.114	−0.077	0.009	1.000

**. Correlation is significant at the 0.01 level (2-tailed). *. Correlation is significant at the 0.05 level (2-tailed).

### Water quality indices

**Fig. 6:**
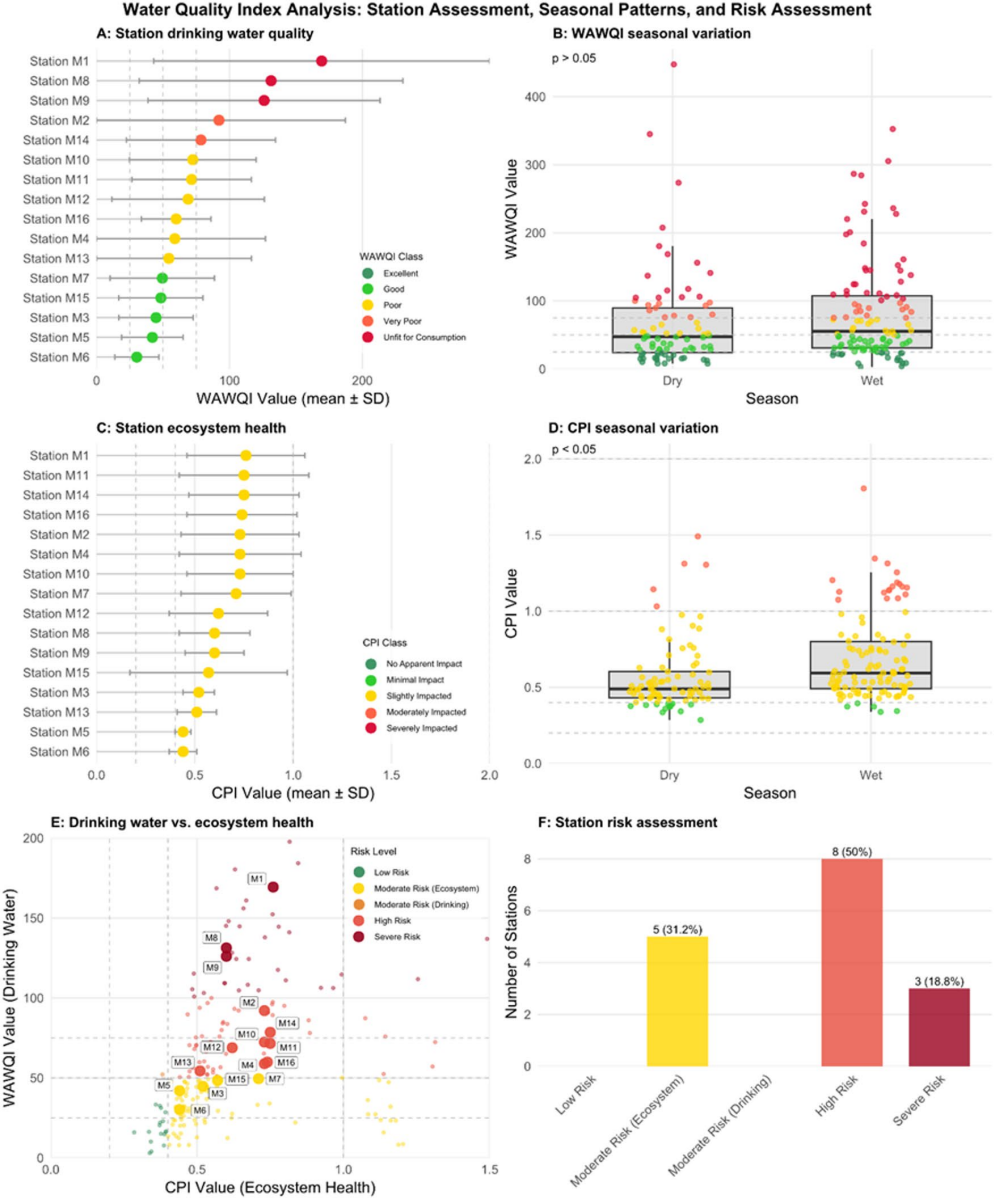
Water quality index analysis for the 16 monitoring stations in the Maziba. (**A**) Mean Weighted Arithmetic Water Quality Index (WAWQI) for drinking water suitability by station. Points represent the mean value, and error bars indicate the standard deviation. (**B**) Seasonal variation of WAWQI values, showing individual measurements as jittered points overlaid on boxplots. (**C**) Mean Comprehensive Pollution Index (CPI) for ecosystem health by station, with error bars indicating standard deviation. (**D**) Seasonal variation of CPI values, with statistical significance from a Wilcoxon test noted (*p* < 0.05). (**E**) Correlation plot of WAWQI versus CPI, with individual measurements (small points) and station averages (large, labelled points) coloured by their combined risk level. (**F**) Bar chart showing the distribution of stations across the five integrated risk categories, with the number and percentage of stations in each class. In panel A-E, dashed lines indicate quality thresholds.

The water quality assessment reveals notable spatial and temporal differences across the 16 monitoring stations (Fig. 6). The Weighted Arithmetic Water Quality Index (WAWQI), which indicates the safety of drinking water, showed that station quality ranged from “Excellent” to “Very Poor” based on the average values (Fig. 6A). Although there was a tendency towards higher WAWQI values (poorer quality) during the wet season, this variation was not statistically significant (*p* > 0.05, Fig. 6B). Conversely, the Comprehensive Pollution Index (CPI), which reflects ecosystem health, exhibited a statistically significant increase during the wet season (*p* < 0.05), implying that runoff events probably contribute to ecological deterioration (Fig. 6D).

The integrated risk assessment, which combines both drinking water and ecosystem health indicators, is shown in Fig. 6e. The distribution of individual measurements and station averages across the risk quadrants confirms a strong link between the two indices; stations with high CPI values also have high WAWQI values, clustering in the ‘High Risk’ and ‘Severe Risk’ zones. As a result, none of the 16 stations could be classified as “Low Risk” (Fig. 6F). The most common category was High Risk, with eight stations (50.0%), characterised by the simultaneous decline in both drinking water quality and ecosystem health. An additional five stations (31.2%) were identified as Moderate Risk (Ecosystem), indicating areas where ecosystem health is affected even if drinking water quality remains acceptable. The remaining three stations (18.8%) fell into the Severe Risk category, representing sites in critical decline that require urgent management action. No stations were classified as “Moderate Risk (Drinking)”, indicating that environmental pressures simultaneously harm both ecosystem health and water quality.

**Fig. 7:**
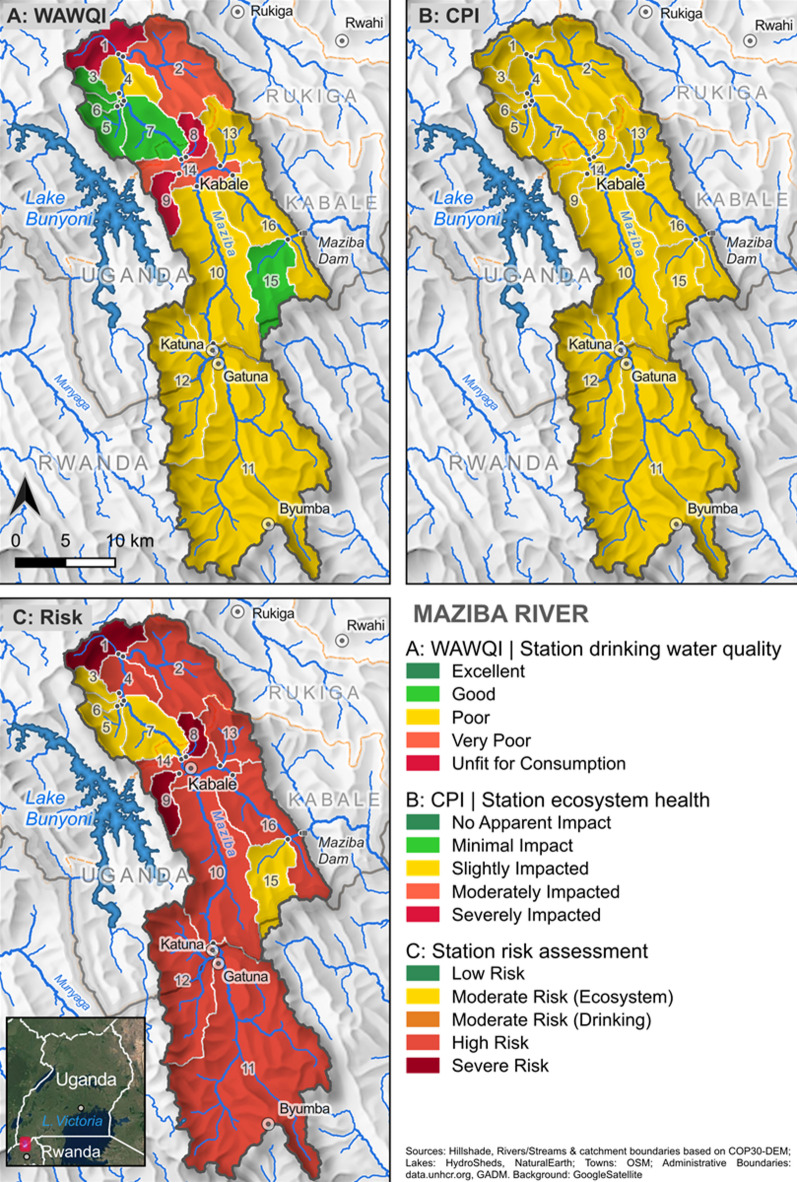
Spatial distribution of water quality indices and risk assessment in the Maziba catchment. (**A**) Weighted Arithmetic Water Quality Index (WAWQI) mapped to sub-catchments upstream of monitoring stations, showing drinking water suitability classifications. The sub-catchments are numbered according to the sampling points.

The spatial mapping of water quality indices to their respective upstream sub-catchments reveals distinct patterns across the Maziba catchment (Fig. 7). The WAWQI distribution indicates that sub-catchments in the northern headwater regions generally exhibit poorer drinking water quality, with the exception of sub-catchments 3, 5, 6, and 7 in the north-west, which show better conditions (Fig. 7A). The CPI mapping indicates that all sub-catchments experience slight ecological impacts (Fig. 7B). The integrated risk assessment demonstrates that no sub-catchments qualify as low risk, with high- to severe-risk areas dominating the catchment (Fig. 7C). This spatial pattern reflects the cumulative effects of land use practices, with urban discharge from Kabale town and intensive agriculture on steep slopes contributing to downstream water quality deterioration.

## Discussion

### Variability in physicochemical parameters

Water temperature showed considerable variation among different sampling stations despite remaining within the ideal range for aquatic organisms, and it does not directly threaten drinking water quality^37^. However, temperatures exceeding 17 °C may promote the survival of pathogens such as *Vibrio cholerae*, as observed in communities vulnerable to cholera in Uganda^38^. The low surface water temperature at Butobere station is caused by the shade provided by nearby riparian vegetation and eucalyptus trees, which reduces sunlight reaching the water. Similarly, Kalny et al. (2017)^39^ found that shaded river reaches cooled downstream by up to 3.5 °C compared to unshaded areas, highlighting the vital role of riparian vegetation in regulating water temperatures. The fluctuating water temperatures have a significant impact on aquatic life, affecting metabolic rates, oxygen levels, and species distribution^40^. They further explain that higher temperatures can increase metabolic demands. If these demands are not met due to insufficient oxygen, sensitive species may experience stress or even mortality.

Dissolved oxygen (DO) levels showed significant differences across study stations. DO levels below 5 mg/L can stress aquatic organisms, indicating increased organic matter decomposition and oxygen use. High DO levels at Ihanga West station suggest minimal organic loading and effective reaeration. Many physical, biochemical, biological, and ecological processes influence DO levels in rivers and streams. These include aeration and diffusion, oxygen production through photosynthesis, oxygen consumption via respiration, organic matter breakdown, and nitrification^41^. The balance between oxygen-consuming processes like organic matter decomposition and replenishing mechanisms such as atmospheric oxygen diffusion and photosynthesis controls DO concentrations in river ecosystems. The highest DO in August 2023 coincided with lower water temperatures, which help retain oxygen. The negative correlation between temperature and DO supports basic physicochemical principles outlined by Ibrahim and Abdulkarim (2017)^42^ in their study on Ajiwa reservoir, confirming that colder waters hold more dissolved oxygen across various stations. Likewise, Saturday et al. (2023)^27^ found significant seasonal changes in DO levels and water temperature in the Lake Mulehe sub-catchment.

The turbidity levels observed ranged from 17.44 ± 7.2 NTU at Ihanga West to 82.38 ± 66.69 NTU at Maziba Dam, both exceeding the WHO-recommended limits for drinking water and aquatic life, which are 5 NTU and 25 NTU, respectively. Maziba Dam Station represents the most downstream study station, where high turbidity levels are mainly linked to surface runoff from agricultural fields and high soil erosion on the steep hillsides upstream. High turbidity can shield harmful microorganisms from disinfection processes and is associated with increased microbial contamination, which poses significant risks to human health. High turbidity levels adversely affect the feeding mechanisms of aquatic life and reduce the photosynthetic efficiency of macrophytes. This relationship is supported by Nimusiima et al. (2023)^25^, who found a significant correlation between high turbidity levels and increased concentrations of *Escherichia coli* and heavy metals in the Kagera River, with turbidity measurements exceeding the WHO guidelines. Furthermore, Sahani (2024)^43^ reported that 93.3% of wetland water sources exhibited turbidity, with only one water source (6.67%) remaining classified as clear (non-turbid) over an 18-month monitoring period in Rukiga District, situated within the Maziba catchment. The pH levels ranged from 6.80 ± 0.24 at Lower Bugongi to 7.30 ± 0.30 at Ihanga West, within the WHO (2018)^37^ recommended range of 6.5–8.5. Although pH alone does not fully determine water quality, these values suggest the water is safe for drinking and supports aquatic life^38^.

Electrical conductivity (EC) showed significant variations across the study stations, with Hakakondogoro and Butobere stations recording the highest levels. These differences reflect the influence of land use on the surrounding areas. Notably, both EC and TDS values remained within the safe limits established by the WHO, indicating that the salinity and mineral content in the water are low. High conductivity levels can be attributed to human activities, mainly from the discharge of domestic wastewater and agricultural runoff, which increase the concentration of dissolved salts and nutrients. These findings align with those of Musungu et al. (2023)^44^, who identified a correlation between high EC values and runoff from tea estates, subsistence farming, and commercial agriculture, particularly during the wet season. Studies near Kampala and Lake Victoria have documented peaks in nitrate levels associated with fertiliser application, particularly the NPK 17:17:17 formulation, and runoff from urban agriculture^45^. Similarly, Njue et al. (2022)^46^ found that significant EC spikes downstream of irrigation schemes in the Thiba River basin correlated with the use of inorganic fertilisers, highlighting the impact of agricultural practices on water quality. The high EC values observed during the dry season are due to concentration effects resulting from lower flow rates, whereas the wet season results in dilution. Likewise, Uwimana et al.(2017)^47^ reported higher EC during dry periods, linking this to farming activities, especially in rice and vegetable cultivation areas in the Migina Catchment, Rwanda. Saturday et al. (2023)^27^ also observed similar results in Lake Mulehe, which they attributed to reduced water flow and increased concentrations of dissolved ions during the dry months.

Total Suspended Solids (TSS) levels varied considerably across the study stations, with Maziba Dam and Katuna exhibiting the highest TSS levels, which far exceeded the usual threshold of 25 to 80 mg/L for healthy freshwater ecosystems^48^. The hilly terrain and poor agricultural practices, including limited vegetation cover, worsen erosion and lead to increased sedimentation in the study area. For instance, high TSS levels can damage benthic habitats and reduce light penetration, hindering photosynthesis in aquatic organisms. Furthermore, elevated TSS can clog fish gills, impairing their ability to breathe and feed. At the Muvumba hydropower dam, sediment accumulation reduces reservoir capacity and impacts hydropower efficiency. The long-term sustainability of this dam may be at risk unless effective measures, such as dredging or upstream restoration, are adopted. The high TSS values observed during the rainy season also indicate ongoing sediment influx due to urban expansion and unpaved roads, which significantly contribute to sedimentation in rivers and streams during rainfalls. In the River Rwizi sub-catchment, Ojok et al. (2017)^49^ noted elevated TSS levels during the rainy season, attributed to soil erosion from agricultural activities and urban runoff, which increases sediment loading in the river. Additionally, increased K^+^ levels are often linked to human waste, as inadequate sanitation practices in Uganda’s peri-urban markets lead to higher potassium concentrations in nearby water bodies.

The nutrient dynamics exhibited complex patterns influenced by hydrological processes and localised nutrient inputs. The highest concentrations of NO₃⁻-N at Brazin Forest and Hakakondogoro were below the WHO limit of 50 mg/L for NO₃⁻ levels^34^. However, this suggests nutrient contributions from untreated sewage and runoff resulting from wastewater channels from nearby homes and agricultural fields that drain into the river system. Elevated NH₄⁺-N levels at Hakakondogoro station may indicate a substantial increase in organic matter from neighbouring animal farms in adjacent sub-watersheds or inadequate nitrification processes due to ineffective ammonium absorption. High levels of SRP, TP, and TN at Maziba Dam and Lower Bugongi stations are associated with household wastewater and agricultural runoff, indicating nutrient enrichment that could lead to algal blooms and eutrophication, thereby negatively impacting water quality and aquatic life. These findings align with Valeriani et al. (2015)^50^, who identified agricultural activities and residential discharges as primary sources of phosphorus in river ecosystems.

Significant variations in nutrient concentrations and seasonal patterns were observed, driven by hydrological and biogeochemical processes. These findings emphasise that transport pathways and biogeochemical processing of nutrient forms can differ notably between wet and dry seasons, affecting water quality management. In December 2023, SRP, NO₃⁻-N, and TP reached their highest levels, while TN peaked in February 2024. These peaks coincided with months of lower flow conditions, typical of the dry season, which likely concentrate both point and non-point nutrient inputs. Saturday et al. (2023)^27^ reported high nutrient levels, particularly SRP, TP, and TN, during the wet season, due to agricultural runoff from the surrounding area in the Lake Mulehe sub-catchment. Furthermore, similar nutrient fluctuations were recorded along the Ugandan stretch of the Kagera Transboundary River, where increased agricultural runoff was linked to elevated NO₃⁻-N levels, underscoring the significant impact of land-use practices on nutrient dynamics in the river system. This variability in nutrient concentrations highlights the need for effective management strategies to mitigate eutrophication risks during periods of nutrient enrichment.

### Correlation analysis of water quality parameters

Principal Component Analysis (PCA) has successfully identified six principal components (PCs) that explain 72.1% of the variance in water quality metrics. The first principal component (PC1) shows a strong correlation with salinity and nutrient pollution. High scores on PC1 indicate increased levels of EC, TDS, and specific ion concentrations (notably Na^+^ and K^+^), as well as higher concentrations of nitrate nitrogen and total nitrogen. Research suggests that in Uganda’s freshwater systems, conductivity values in pristine rivers and lakes generally range from 100 to 500 µS/cm^38^. In contrast, Ling et al. (2017)^51^ identified six components accounting for 83.6% of the variance in their analysis, with primary pollutants linked to logging activities being total suspended solids, turbidity, and hydrogen sulfide. Their second component was associated with domestic pollution indicators, such as biochemical oxygen demand and phosphorus levels.

The Spearman correlation analysis revealed significant relationships among various water quality parameters. Temperature showed a significant negative correlation with DO levels, demonstrating that oxygen solubility decreases as temperatures rise. This pattern is supported by Szewczyk et al. (2023)^52^, who observed that higher water temperatures in the Waccamaw River in the Pee Dee Basin of the Southeastern United States led to lower DO levels. Additionally, a notable negative correlation was found between TSS and Chl-a concentrations. This suggests that turbidity levels increase as suspended sediments from surface runoff accumulate. The increased turbidity likely reduces light penetration in the water, thereby hampering photosynthesis and primary productivity. These results emphasise the complex interconnection of physical, chemical, and biological processes, all of which play a vital role in affecting water quality in our study area. Phosphorus species, including SRP and TP, correlated strongly with pH, EC, TDS, and major ions such as Na⁺, K⁺, and Cl⁻. These findings indicate that agricultural and municipal runoff are the main sources of phosphorus. This enrichment probably results from the use of fertilisers and urban effluents, which increase ion concentrations and phosphates. Nitrogen species, such as NO₂⁻-N, NH₄⁺-N, and TN, showed strong positive correlations with turbidity, chlorophyll-a, EC, and TDS. This reflects the impact of nutrient enrichment from fertilisers and organic matter on algal growth and particulate matter. Similarly, Umuhoza et al. (2024)^53^ reported that TN and TP were strongly associated with turbidity, EC, and TDS in the Nyabarongo River of Rwanda.

While anthropogenic activities are the primary drivers of water quality degradation in the Maziba catchment, suspended sediments may contribute to pollutant mobilization from underlying geology. Elevated TSS concentrations during the wet season indicate substantial erosion, with suspended particles adsorbing and transporting geogenic contaminants such as heavy metals and trace elements from the metamorphic and sedimentary rocks of southwestern Uganda.

### Water quality indices and their implications

The Weighted Arithmetic Water Quality Index (WAWQI) indicated that most stations (69%) had water quality unsuitable for drinking, with classifications ranging from “poor” to “unfit.” A notable contrast was observed in headwater catchments: while four such stations exhibited “excellent” or “good” quality, reflecting cleaner conditions, three others were considered “unfit.” This significant degradation is attributed to specific anthropogenic pressures, namely urban influences from Kabale town affecting Lower Bugongi (M8) and Brazin Forest (M9) stations, and intensive agriculture impacting Hakakondogoro station (M1).

The WAWQI generally shows significant temporal and spatial differences in drinking water quality across various study locations. These differences highlight the important influence of local factors, especially urban wastewater discharges and agricultural runoff, on the physicochemical properties of water. For example, Sanusi et al. (2024)^54^ found that during the wet season in the Kampala/Mbarara region, the proportion of sampling sites classified as “excellent” decreased sharply, with some sites deemed unsuitable for consumption due to high pollutant levels. Similarly, Saturday et al.(2021)^26^ reported that the lower catchment areas of Lake Bunyonyi consistently had lower WQI scores compared to upstream sites. Their findings showed that wastewater discharge from trading centres and agricultural runoff increased nutrient and contaminant levels in downstream locations^26^. These observations demonstrate that urban and peri-urban areas, along with downstream sites, often show higher pollution indices, emphasising the usefulness of WAWQI in capturing spatial differences and seasonal changes in tropical watersheds^54^.

The variations in water quality are further affected by urban stormwater runoff and rainfall-induced discharge. Intense tropical rainfall tends to mobilise nutrients and pollutants from land surfaces into nearby water bodies. For example, satellite monitoring over Lake Victoria has shown that increased rainfall correlates with greater erosion and nutrient transport (including nitrogen and phosphorus) into the lake, promoting algal blooms and increased turbidity^45^. Besides the impacts of natural rainfall, urbanisation and agricultural expansion worsen these issues. Urban runoff, along with poor sanitation, often introduces untreated sewage and agrochemicals into water bodies, while farming practices contribute to the runoff of fertilisers and pesticides. Research indicates that runoff from suburban Kampala during rainy periods, along with discharges from markets, leads to rapid increases in WQI values^54^. Interestingly, our data and the WAWQI display a slight decline in water quality during the rainy season. However, the differences are not statistically significant.

The CPI evaluated water suitability for aquatic life, showing that it was, on average, “slightly impacted” across the study stations. Seasonal analysis revealed greater variability, with results ranging from “minimal impact” to “moderately impacted”. These findings indicate that, despite human pressures such as farming activities, the ecological health of aquatic environments generally remains relatively stable. Similarly, Mekonnen and Tekeba (2024) observed that the Shinta River in Ethiopia, affected by both brewery and municipal waste, was rated as merely “minimal impacted” (CPI around 0.2–0.4) in its upper reaches but showed moderate to severe pollution levels (CPI approximately 0.8–5.0) downstream, affecting drinking water and aquatic life^8^. Seasonal fluctuations amplify these patterns: during the rainy season, runoff transports nutrients and organic materials into rivers, worsening pollution metrics. For example, Chen et al. (2022)^55^ discovered that during the wet season in Mwanza Gulf (Lake Victoria), agricultural runoff and urban effluents led to elevated nitrogen and phosphorus levels, resulting in the lowest water quality index (WQI) scores.

Overall, the results of this study agree with findings from both tropical and global research, showing that human activities (such as urban wastewater and fertilisers) cause higher WAWQI/CPI scores, which indicate poorer water quality, especially during heavy rain. For example, Podlasek et al. (2025)^56^ reported average WQI values between 63 and 97 and CPI values from 0.56 to 0.88 in surface waters affected by landfills, which usually signal good water quality. Combining index methods effectively monitors changes in water quality over time and across locations, highlighting the need to control pollution from urban and agricultural sources to improve index ratings and protect ecosystems.

The integrated risk assessment offers a holistic insight into the nature of environmental pressure within the Maziba catchment. The complete absence of any “low-risk” stations underscores that the entire monitored system is affected by water quality degradation. The strong linkage observed between the CPI and WAWQI values, with a majority of stations classified as “High Risk” (50.0%) or “Severe Risk” (18.8%), indicates that the sources of pollution are not specialised. Instead, they appear to be degrading the water from both ecological and human-use perspectives simultaneously, a characteristic of mixed-contaminant sources, namely agricultural and urban runoff. No stations are classified as “Moderate Risk for drinking”. This finding suggests that in the Maziba system, ecological health is not a standalone early warning indicator that degrades long before drinking water suitability is compromised. The environmental pressures are such that by the time ecological indicators decline, parameters relevant to human consumption are already, or are simultaneously, impacted. This concurrent degradation implies that management strategies must be integrated to address the issue effectively. Interventions aimed solely at ecosystem restoration or at improving drinking water quality in isolation may be inefficient; instead, actions that reduce agricultural runoff, manage urban wastewater, and control erosion are required to improve the overall health of the watershed and safeguard both ecosystem functions and public health.

## Conclusion

This study provides a detailed evaluation of water quality across sixteen watersheds in the Maziba sub-catchment, using monthly monitoring data from July 2023 to June 2024 to examine spatiotemporal variations. The findings reveal significant ionic and nutrient enrichment, with Principal Component Analysis indicating that six components account for 72.1% of the total variance. The first component confirms that pollution is primarily driven by salinity and nutrient indicators (EC, TDS, Na⁺, Cl⁻, K⁺, NO₃⁻-N), which are linked to agricultural runoff and urban wastewater, especially during the rainy seasons. The application of the Weighted Arithmetic Water Quality Index (WAWQI) and the Comprehensive Pollution Index (CPI) simplifies complex data into understandable values, facilitating communication of water quality to stakeholders. The assessment highlights a critical situation in Maziba: the WAWQI classifies 69% of the stations as having water that is “poor” to “unfit” for drinking. The combined risk assessment corroborates this, categorising most sites as “High Risk” (50.0%) or “Severe Risk” (18.8%), with no stations falling into the “Low Risk” category. The framework reveals a concurrent decline in drinking water quality and ecosystem health, with the direct implication for water management being that interventions must be integrated. Strategies should comprehensively address both diffuse agricultural runoff and urban pollution to improve the catchment’s condition. Furthermore, this study addresses the regional issue of data scarcity by making the complete dataset freely accessible to support future research and monitoring efforts.

This study has limitations affecting the interpretation of results. Excluding microbiological indicators, such as *E. coli*, prevents the assessment of faecal contamination and associated health risks. The focus on physicochemical parameters may overlook contaminants such as pesticides or emerging pollutants. A one-year monitoring period limits understanding of long-term trends and seasonal patterns. Results are from specific sites in the Kigezi Highlands and may not represent wider conditions due to site-specific factors and hydrological variability. The WAWQI/CPI and risk assessment methods simplify complex data, potentially obscuring individual pollutant effects. Future research should include microbiological tests, longer monitoring periods, and broader spatial coverage to assess water quality in tropical agricultural catchments better.

## Data Availability

Data is published online available at https://doi.org/10.5281/zenodo.15465720.

## Appendices

### Appendix A1: Mean ± SD of physicochemical parameters at different study stations (n= 192)

**Table:**

Station ID	Station	WT (°C)	DO (mg/L)	Tur (NTU)	EC (µS/cm)	TSS (mg/L)	Chl-a (µg/L)
M1	Hakakondogoro	19.48 ± 3.24	4.36 ± 2.03	55.70 ± 51.78	233.75 ± 40.96	103.67 ± 88.46	5.75 ± 2.51
M2	Kakore East	19.37 ± 1.78	4.53 ± 1.67	72.49 ± 67.38	233.25 ± 36.35	137.25 ± 14.08	6.10 ± 2.48
M3	Mukirwa	19.91 ± 3.94	6.47 ± 1.89	30.50 ± 19.70	214.33 ± 72.29	145.67 ± 219.35	2.88 ± 1.14
M4	Ihanga FGC	19.23 ± 2.19	5.55 ± 1.10	80.22 ± 76.14	212.92 ± 72.03	182.25 ± 108.27	6.55 ± 2.85
M5	Ihanga	19.33 ± 2.04	6.58 ± 0.79	17.44 ± 7.22	162.83 ± 43.26	92.42 ± 87.36	2.19 ± 0.98
M6	Ihanga West	19.17 ± 3.31	6.91 ± 1.15	17.84 ± 11.34	188.75 ± 61.27	52.42 ± 30.99	1.95 ± 0.63
M7	Rwakaraba	19.71 ± 2.24	6.08 ± 1.11	75.64 ± 68.05	220.92 ± 72.65	175.33 ± 174.08	5.70 ± 2.19
M8	Lower Bugongi	20.57 ± 1.90	4.31 ± 1.44	27.01 ± 15.24	246.17 ± 88.10	83.83 ± 38.02	3.48 ± 1.26
M9	Brazin Forest	19.98 ± 1.41	5.48 ± 1.30	26.98 ± 13.07	206.00 ± 48.43	70.58 ± 21.23	3.07 ± 0.78
M10	Rugyendira	20.63 ± 1.57	5.48 ± 0.94	77.74 ± 55.71	156.83 ± 59.90	198.75 ± 158.01	5.58 ± 1.61
M11	Muvumba	21.43 ± 2.32	5.65 ± 1.04	79.34 ± 67.03	155.83 ± 56.60	223.58 ± 297.33	4.64 ± 2.19
M12	Katuna	22.15 ± 2.65	5.15 ± 1.89	51.09 ± 43.12	198.00 ± 79.60	287.75 ± 288.54	3.80 ± 1.77
M13	Butobere	18.27 ± 1.43	5.59 ± 0.72	28.83 ± 8.48	262.25 ± 74.99	118.00 ± 72.16	3.10 ± 0.55
M14	Kyanamira	19.82 ± 1.42	5.88 ± 1.16	78.01 ± 61.80	177.58 ± 34.02	260.67 ± 250.61	5.91 ± 1.14
M15	Kabanyonyi	19.15 ± 2.66	6.07 ± 1.10	51.44 ± 85.94	73.50 ± 23.72	90.75 ± 32.53	2.86 ± 0.52
M16	Maziba Dam	20.95 ± 2.60	5.48 ± 1.47	82.38 ± 66.69	163.50 ± 38.51	298.75 ± 343.81	5.75 ± 1.45

### Appendix A2: Mean ± SD of physicochemical parameters at different study stations (n= 192) (Cont’d)

**Table:**

Station ID	Stations	PH	EC (µS/cm)	TDS (mg/L)	TH (mg/L)	Na^+^ (mg/L)	Cl (mg/L)	SO_4_ (mg/L)	K^+^(mg/L)
M1	Hakakondogoro	6.84 ± 0.30	233.75 ± 40.96	163.58 ± 28.69	91.17 ± 12.11	11.00 ± 3.46	20.90 ± 8.66	29.47 ± 16.23	3.98 ± 1.23
M2	Kakore East	6.97 ± 0.18	233.25 ± 36.35	163.29 ± 25.48	88.75 ± 15.96	10.89 ± 1.39	21.74 ± 4.65	28.59 ± 11.43	4.03 ± 1.22
M3	Mukirwa	7.28 ± 0.25	214.33 ± 72.29	150.04 ± 50.60	82 ± 15.10	9.93 ± 3.21	24.34 ± 10.82	35.08 ± 12.61	3.25 ± 0.91
M4	Ihanga FGC	6.92 ± 0.24	212.92 ± 72.03	149.05 ± 50.43	89.17 ± 14.67	9.66 ± 2.90	19.57 ± 6.69	31.19 ± 15.24	3.76 ± 1.28
M5	Ihanga	6.99 ± 0.30	162.83 ± 43.26	113.88 ± 30.23	67.58 ± 11.18	6.86 ± 1.52	17.18 ± 6.13	27.64 ± 13.30	2.49 ± 0.28
M6	Ihanga West	7.30 ± 0.30	188.75 ± 61.27	132.10 ± 42.88	76.08 ± 9.73	8.43 ± 2.64	23.49 ± 10.34	29.99 ± 11.25	3.29 ± 0.46
M7	Rwakaraba	7.06 ± 0.15	220.92 ± 72.65	154.6 ± 50.82	86.25 ± 16.22	10.53 ± 3.27	20.65 ± 8.03	37.00 ± 23.38	3.49 ± 1.20
M8	Lower Bugongi	6.80 ± 0.24	246.17 ± 88.10	172.28 ± 61.65	73.67 ± 12.92	14.60 ± 7.69	26.94 ± 10.97	26.05 ± 6.56	7.46 ± 4.43
M9	Brazin Forest	6.96 ± 0.14	206.00 ± 48.43	152.87 ± 11.49	69.25 ± 19.13	10.82 ± 1.38	21.39 ± 4.30	25.69 ± 6.78	4.45 ± 0.79
M10	Rugyendira	6.85 ± 0.20	156.83 ± 59.90	109.77 ± 41.93	64.83 ± 16.84	7.07 ± 2.87	14.46 ± 5.83	19.04 ± 9.47	3.32 ± 1.99
M11	Muvumba	6.93 ± 0.17	155.83 ± 56.60	100.46 ± 41.99	64.50 ± 21.00	7.65 ± 4.53	17.48 ± 9.68	18.12 ± 6.43	2.88 ± 1.23
M12	Katuna	6.80 ± 0.53	198.00 ± 79.60	138.60 ± 55.72	60.08 ± 26.27	10.68 ± 4.82	22.91 ± 10.06	21.65 ± 8.05	3.85 ± 1.49
M13	Butobere	6.94 ± 0.22	262.25 ± 74.99	183.54 ± 52.47	82.00 ± 11.09	15.22 ± 5.06	31.73 ± 12.71	34.38 ± 9.51	4.98 ± 1.67
M14	Kyanamira	6.91 ± 0.36	177.58 ± 34.02	124.25 ± 23.77	69.75 ± 13.77	9.22 ± 2.44	18.68 ± 3.93	25.57 ± 11.28	3.84 ± 1.59
M15	Kabanyonyi	6.87 ± 0.29	73.50 ± 23.72	51.44 ± 16.60	53.58 ± 19.34	2.68 ± 0.53	4.37 ± 1.83	5.18 ± 3.06	1.51 ± 0.29
M16	Maziba Dam	7.19 ± 0.37	163.50 ± 38.51	114.43 ± 26.93	71.08 ± 6.17	7.58 ± 1.67	16.89 ± 4.86	23.60 ± 10.52	3.08 ± 0.57

### Appendix A3: Mean ± SD of physicochemical parameters at different study stations (Cont’d)

**Table:**

Station ID	Stations	TP (mg/L)	TN (mg/L)	SRP (mg/L)	NO₂⁻-*N* (mg/L)	NH₄⁺-*N*(mg/L)	NO₃⁻-*N* (mg/L)
M1	Hakakondogoro	0.30 ± 0.26	3.62 ± 1.11	0.08 ± 0.05	0.12 ± 0.20	0.41 ± 0.32	1.91 ± 0.74
M2	Kakore East	0.34 ± 0.28	3.66 ± 0.66	0.11 ± 0.10	0.09 ± 0.05	0.22 ± 0.24	2.16 ± 0.70
M3	Mukirwa	0.21 ± 0.14	3.68 ± 0.74	0.06 ± 0.05	0.01 ± 0.01	0.10 ± 0.07	2.68 ± 0.90
M4	Ihanga FGC	0.26 ± 0.21	3.39 ± 0.90	0.08 ± 0.08	0.04 ± 0.03	0.13 ± 0.17	2.16 ± 0.79
M5	Ihanga	0.18 ± 0.16	3.43 ± 0.71	0.05 ± 0.07	0.02 ± 0.01	0.10 ± 0.06	2.60 ± 0.77
M6	Ihanga West	0.16 ± 0.19	3.60 ± 1.07	0.05 ± 0.03	0.01 ± 0.02	0.07 ± 0.04	2.79 ± 1.11
M7	Rwakaraba	0.23 ± 0.21	3.13 ± 0.84	0.07 ± 0.05	0.03 ± 0.03	0.11 ± 0.10	2.12 ± 0.67
M8	Lower Bugongi	0.23 ± 0.17	5.03 ± 1.71	0.11 ± 0.08	0.11 ± 0.12	0.32 ± 0.25	3.12 ± 1.14
M9	Brazin Forest	0.28 ± 0.33	4.91 ± 0.87	0.11 ± 0.19	0.08 ± 0.09	0.30 ± 0.22	3.46 ± 0.78
M10	Rugyendira	0.28 ± 0.27	3.78 ± 0.76	0.06 ± 0.06	0.11 ± 0.27	0.17 ± 0.12	2.59 ± 0.38
M11	Muvumba	0.19 ± 0.16	3.93 ± 0.93	0.06 ± 0.05	0.07 ± 0.11	0.17 ± 0.11	2.64 ± 0.67
M12	Katuna	0.21 ± 0.22	3.99 ± 1.77	0.06 ± 0.05	0.04 ± 0.03	0.16 ± 0.14	2.51 ± 1.57
M13	Butobere	0.12 ± 0.09	4.33 ± 1.12	0.03 ± 0.02	0.02 ± 0.02	0.13 ± 0.16	3.01 ± 1.31
M14	Kyanamira	0.40 ± 0.46	3.94 ± 1.40	0.08 ± 0.08	0.05 ± 0.05	0.18 ± 0.14	2.18 ± 1.10
M15	Kabanyonyi	0.11 ± 0.08	2.95 ± 0.88	0.04 ± 0.03	0.04 ± 0.07	0.11 ± 0.08	1.93 ± 0.75
M16	Maziba Dam	0.40 ± 0.57	4.52 ± 3.47	0.08 ± 0.08	0.07 ± 0.12	0.13 ± 0.07	2.33 ± 0.96

### Appendix A4: Mean ± SD of physicochemical parameters across sampling months (n= 192)

**Table:**

Months	WT (°C)	DO (mg/L)	Turbidity (NTU)	Chl-a (µg/L)	pH	EC (µS/cm)	TDS (mg/L)
Aug 2023	17.87 ± 1.89	7.41 ± 0.72	26.04 ± 12.84	3.00 ± 1.20	7.38 ± 0.21	199.75 ± 61.82	139.83 ± 43.27
Sept 2023	19.10 ± 2.66	6.26 ± 1.88	43.12 ± 22.53	4.72 ± 1.74	7.04 ± 0.26	225.69 ± 74.65	157.88 ± 52.22
Oct 2023	21.20 ± 2.99	6.05 ± 0.78	69.82 ± 61.29	4.74 ± 2.92	6.95 ± 0.24	128.31 ± 75.73	89.80 ± 53.00
Nov 2023	19.23 ± 2.49	5.98 ± 1.20	106.79 ± 74.48	5.39 ± 2.65	6.87 ± 0.19	221.06 ± 66.97	154.74 ± 46.88
Dec 2023	19.22 ± 2.44	5.89 ± 1.21	107.78 ± 74.84	5.94 ± 2.65	6.87 ± 0.19	102.75 ± 57.16	71.93 ± 40.01
Jan 2024	18.97 ± 0.81	5.30 ± 1.79	58.53 ± 61.54	4.79 ± 2.35	7.21 ± 0.21	176.38 ± 48.00	123.44 ± 33.58
Feb 2024	19.75 ± 2.56	5.23 ± 1.09	78.81 ± 50.18	6.16 ± 2.37	6.89 ± 0.55	234.75 ± 56.92	164.33 ± 39.85
Mar 2024	21.44 ± 2.17	5.04 ± 1.10	33.09 ± 17.06	4.09 ± 1.43	7.07 ± 0.27	177.00 ± 69.99	123.90 ± 48.99
Apr 2024	20.87 ± 1.55	4.89 ± 1.23	27.32 ± 9.02	3.91 ± 1.54	6.91 ± 0.23	203.06 ± 52.93	142.14 ± 7.05
May 2024	21.36 ± 1.86	4.75 ± 1.18	56.07 ± 72.65	4.48 ± 1.76	6.84 ± 0.19	228.81 ± 62.11	160.17 ± 43.48
June 2024	19.53 ± 2.29	5.37 ± 1.33	19.80 ± 5.69	2.83 ± 0.80	6.88 ± 0.23	216.06 ± 55.75	151.18 ± 39.07
Jul 2024	20.71 ± 3.02	4.91 ± 1.90	13.33 ± 5.72	2.50 ± 1.10	6.83 ± 0.30	216.19 ± 58.44	151.33 ± 40.91
**All Grps**	**19.95 ± 2.50**	**5.60 ± 1.49**	**53.29 ± 55.79**	**4.33 ± 2.21**	**6.98 ± 0.31**	**194.15 ± 72.38**	**135.89 ± 50.66**

### Appendix A5: Mean ± SD of physicochemical parameters across study months (continued)

**Table:**

Months	TH mg/L	Na^+^ (mg/L)	K^+^ (mg/L)	Cl⁻ (mg/L)	SO₄²⁻(mg/L)	TSS (mg/L)
Aug 2023	76.13 ± 16.54	12.13 ± 6.65	3.71 ± 2.07	22.70 ± 9.38	23.38 ± 10.47	144.88 ± 120.57
Sept 2023	76.56 ± 26.18	10.54 ± 4.44	3.63 ± 1.57	21.66 ± 8.98	39.13 ± 17.76	103.94 ± 53.23
Oct 2023	84.63 ± 11.89	6.58 ± 3.22	2.94 ± 1.35	13.16 ± 9.91	18.21 ± 11.77	161.50 ± 133.27
Nov 2023	76.13 ± 16.54	10.59 ± 4.54	4.35 ± 1.57	23.59 ± 8.95	32.11 ± 10.44	353.38 ± 308.84
Dec 2023	76.56 ± 26.18	5.70 ± 3.65	2.64 ± 0.81	8.91 ± 5.09	39.13 ± 17.76	106.63 ± 48.00
Jan 2024	72.38 ± 26.89	8.33 ± 2.80	3.36 ± 1.36	19.63 ± 8.91	16.93 ± 6.54	208.56 ± 361.83
Feb 2024	67.25 ± 18.74	10.66 ± 4.57	6.37 ± 3.93	22.05 ± 7.53	28.56 ± 7.82	275.19 ± 283.37
Mar 2024	75.25 ± 6.85	8.80 ± 4.07	2.97 ± 1.48	19.20 ± 9.43	20.97 ± 9.45	113.00 ± 49.76
Apr 2024	69.00 ± 21.27	10.71 ± 4.28	3.36 ± 1.31	21.56 ± 8.30	20.61 ± 8.49	117.13 ± 46.12
May 2024	70.31 ± 14.28	9.28 ± 3.53	4.10 ± 1.66	22.13 ± 9.33	19.61 ± 7.44	95.56 ± 92.88
June 2024	68.00 ± 13.59	10.71 ± 3.86	3.58 ± 1.29	24.51 ± 10.04	34.25 ± 15.46	112.19 ± 91.90
Jul 2024	80.13 ± 10.68	10.58 ± 3.70	3.74 ± 1.15	22.96 ± 9.16	20.79 ± 7.50	99.31 ± 73.04
**All Grps**	**74.36 ± 18.70**	**9.55 ± 4.47**	**3.73 ± 1.97**	**20.17 ± 9.66**	**26.14 ± 13.68**	**157.60 ± 187.84**

### Appendix A6: Mean ± SD of physicochemical parameters across sampling months

**Table:**

Months	NO₂⁻-*N* (mg/L)	NH₄⁺-*N* (mg/L)	SRP (mg/L)	NO₃⁻-*N* (mg/L)	TN (mg/L)	TP (mg/L)
Aug 2023	0.20 ± 0.29	0.09 ± 0.06	0.02 ± 0.01	2.60 ± 1.28	3.24 ± 0.91	0.09 ± 0.06
Sept 2023	0.04 ± 0.03	0.31 ± 0.23	0.05 ± 0.07	2.31 ± 0.60	3.94 ± 0.78	0.06 ± 0.09
Oct 2023	0.03 ± 0.03	0.14 ± 0.17	0.05 ± 0.07	1.75 ± 0.72	2.45 ± 1.03	0.06 ± 0.09
Nov 2023	0.11 ± 0.12	0.16 ± 0.10	0.07 ± 0.05	3.10 ± 0.57	4.40 ± 0.86	0.34 ± 0.33
Dec 2023	0.04 ± 0.06	0.13 ± 0.20	0.03 ± 0.01	1.37 ± 0.53	3.99 ± 0.81	0.04 ± 0.02
Jan 2024	0.03 ± 0.02	0.23 ± 0.13	0.06 ± 0.03	2.45 ± 0.59	4.02 ± 0.70	0.22 ± 0.13
Feb 2024	0.02 ± 0.02	0.18 ± 0.15	0.09 ± 0.05	2.80 ± 0.74	5.79 ± 2.85	0.47 ± 0.29
Mar 2024	0.03 ± 0.04	0.19 ± 0.16	0.08 ± 0.05	2.12 ± 1.26	2.93 ± 1.14	0.23 ± 0.17
Apr 2024	0.05 ± 0.06	0.11 ± 0.09	0.05 ± 0.04	2.33 ± 0.83	3.23 ± 0.84	0.15 ± 0.11
May 2024	0.04 ± 0.03	0.29 ± 0.18	0.07 ± 0.07	3.16 ± 1.13	4.62 ± 0.81	0.34 ± 0.15
June 2024	0.03 ± 0.03	0.13 ± 0.20	0.18 ± 0.14	3.60 ± 0.61	4.43 ± 0.76	0.26 ± 0.17
Jul 2024	0.03 ± 0.03	0.14 ± 0.27	0.10 ± 0.09	2.56 ± 0.58	3.39 ± 0.84	0.65 ± 0.46
**All Grps**	**0.06 ± 0.11**	**0.18 ± 0.18**	**0.07 ± 0.08**	**2.51 ± 1.00**	**3.87 ± 1.43**	**0.24 ± 0.27**
